# Circulating Gremlin-1 is elevated in systemic sclerosis patients

**DOI:** 10.1177/23971983211036571

**Published:** 2021-08-02

**Authors:** Steven O’Reilly

**Affiliations:** Department of Biosciences, Durham University, Durham, UK

**Keywords:** Gremlin-1, bone morphogenetic protein, lung fibrosis, scleroderma, transforming growth factor-beta

## Abstract

**Introduction:**

Systemic sclerosis is an autoimmune connective tissue disease in which there is activation of the immune system, vascular disease and fibrosis. Activation of quiescent fibroblasts to myofibroblasts is key to disease pathogenesis. Gremlin-1 is a bone morphogenetic protein antagonist which is important in development and we recently reported in skin fibrosis. The aim of this study was to determine the serum circulating levels of Gremlin-1 in early diffuse systemic sclerosis.

**Methods:**

Twenty-one early diffuse systemic sclerosis patients (less than 2 years from first non-Raynaud’s symptom) were included and age and sex-matched healthy controls. Serum was isolated from blood and measured with a specific enzyme-linked immunoassay for Gremlin-1. Clinical variables were also measured.

**Results:**

Significantly elevated Gremlin-1 was found in sera of early diffuse systemic sclerosis patients (*p* < 0.001). In patients with interstitial lung disease, this compared to systemic sclerosis without evidence of interstitial lung disease, Gremlin-1 was significantly elevated (*p* < 0.0007). A correlation was found between circulating Gremlin-1 and modified Rodnan Skin Score, albeit weak.

**Discussion:**

In early diffuse systemic sclerosis patients, elevated Gremlin-1 is found in serum. This is particularly prominent in systemic sclerosis–associated interstitial lung disease. This suggests that Gremlin-1 may be a biomarker for systemic sclerosis interstitial lung disease.

## Introduction

Systemic sclerosis (SSc) is an idiopathic autoimmune connective tissue disease which is characterised by vascular abnormalities, inflammation and fibrosis.^1,2^ The disease is likely initiated by a vascular injury, leading to inflammation and finally fibrosis. The fibrosis primarily affects the skin and can affect the lungs.^3^ The extent of skin thickening and organ involvement can delineate this disease into two subtypes: limited cutaneous SSc in which only limited to discrete areas of the skin and diffuse SSc in which there is larger involvement of the skin and internal organs. Currently, no treatment is licenced for the skin fibrotic element of the disease, although recently treatments have been licenced for lung disease associated with SSc, such as nintedanib. Activation of quiescent fibroblasts to activated myofibroblasts that secrete copious amounts of extracellular matrix is at the core of the disease, and understanding of how these cells are activated remains elusive.

Gremlin-1 is a bone morphogenetic protein (BMP) antagonist that is required for embryogenesis.4 Gremlin-1 is part of the transforming growth factor (TGF)-β1 superfamily and acts to block by sequestering soluble BMPs. BMPs have to be tightly regulated, and thus, Gremlin-1 is one such protein that regulates this.^4,5^ Recently, Gremlin-1 has been found to be associated with kidney,^6,7^ lung^8,9^ and skin fibrosis.^10^ We described very recently that overexpression of Gremlin-1 led to increased myofibroblast transition that was partially dependant on TGF-β1 signalling, as blockade of TGF-β1 mitigated fibrosis.^11^ Analysis of SSc fibroblasts compared to controls did not find elevated levels of Gremlin-1. The aim of this study is to determine the levels of Gremlin-1 in the sera of SSc patients with early diffuse SSc disease.

## Methods

Twenty-one patients with early diffuse SSc were involved in the study; this is a retrospective study in a single-centre study. Patients were defined as early diffuse SSc defined as <2 years since the first non-Raynaud’s symptom. All patients fulfilled the American College of Rhuematology (ACR) criteria for a diagnosis of diffuse SSc and full informed consent was provided. The study has full ethical approval with the local research ethics committee (REC) with approval no. REC/13/NE/0089 and followed the declaration of Helsinki guidelines. Healthy controls (HCs) were age and gender matched (*n* = 20). 15 mL blood was drawn from each donor arm, and serum was isolated by centrifugation at 2000*g* for 15 min.

## ELISA

A commercially available enzyme-linked immunosorbent assay (ELISA) was used purchased from Reagent Genie (Dublin, Republic of Ireland). This recognises human Gremlin-1. A standard curve was constructed from known amounts of Gremlin-1, and data were calculated from this curve.

Data were tested for normality using the Kolmogorov–Smirnov test of normality. Normal distribution as determined by the Kolmogorov–Smirnov test, Student’s *t*-test was undertaken. Student’s *t*-test was used to test differences between HC and SSc patients with a *p*-value < 0.05 considered statistically significant. The Mann–Whitney *U* test was used to compare differences between SSc without lung disease and those with. For correlation analysis, Pearson’s correlation *r* value was calculated using Prism™ software package.

## Results

Twenty-one early diffuse SSc patients were included in this study with 20 HCs. Table 1 gives the patients’ demographics. Of the SSc patients, 5 had interstitial lung disease (ILD) and 16 did not have any evidence of ILD. Diffusing capacity of the lung for carbon monoxide (D_LCO_)% predicted of 60% or less was used to define ILD. No patient was receiving any treatment medications.

**Table 1.** SSc diffuse patient demographics.

Patient number	Age (years)	Sex	Autoantibodies	mRSS (1)	Treatment	ILD	D_LCO_%
Patient_1	48	F	Scl-70	9	None	N	85
Patient_2	54	F	Scl-70	16	None	N	82
Patient_3	51	F	RNA-polIII	10	None	N	89
Patient_4	66	F	Scl-70	16	None	Y	60
Patient_5	39	F	Scl-70	11	None	N	73
Patient_6	52	F	Scl-70	12	None	N	79
Patient_7	41	M	Scl-70	10	None	N	87
Patient_8	49	F	Scl-70	14	None	Y	57
Patient_9	55	F	RNA-polIII	17	None	Y	52
Patient_10	42	F	Scl-70	21	None	N	91
Patient_11	57	M	Scl-70	14	None	N	75
Patient_12	35	F	Scl-70	19	None	N	82
Patient_13	47	F	Scl-70	11	None	N	76
Patient_14	61	F	Scl-70	15	None	N	74
Patient_15	55	F	Scl-70	21	None	Y	50
Patient_16	37	F	Scl-70	18	None	N	78
Patient_17	48	F	Scl-70	22	None	Y	52
Patient_18	51	F	Scl-70	16	None	N	77
Patient_19	39	F	RNA-polIII	12	None	N	93
Patient_20	42	F	Scl-70	26	None	N	70
Patient_21	37	M	Scl-70	19	None	N	74

ILD: interstitial lung disease; D_LCO_: carbon monoxide lung diffusion capacity % predicted.

1 mRSS is the modified Rodnan Skin Score based on 1–51.

**Figure 1.**
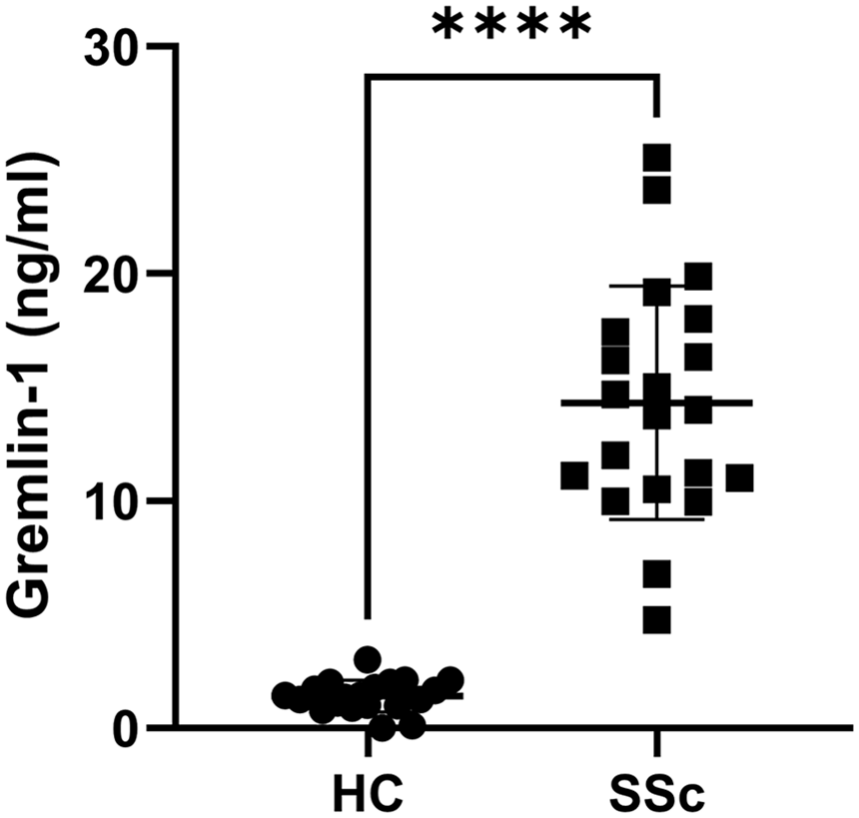
Elevated Gremlin-1 in SSc patients. Gremlin-1 was quantified by specific ELISA. Data are each individual point represent a single donor. ***p < 0.0001 (Student’s *t*-test).

Gremlin-1 levels in SSc were significantly higher compared to HCs (Figure 1): mean = 1.14 ng/mL (standard deviation (SD) = 0.7) versus mean = 14.32 ng/mL (SD = 5.1), *n* = 21, *p* < 0.0001; Student’s *t*-test.

**Figure 2.**
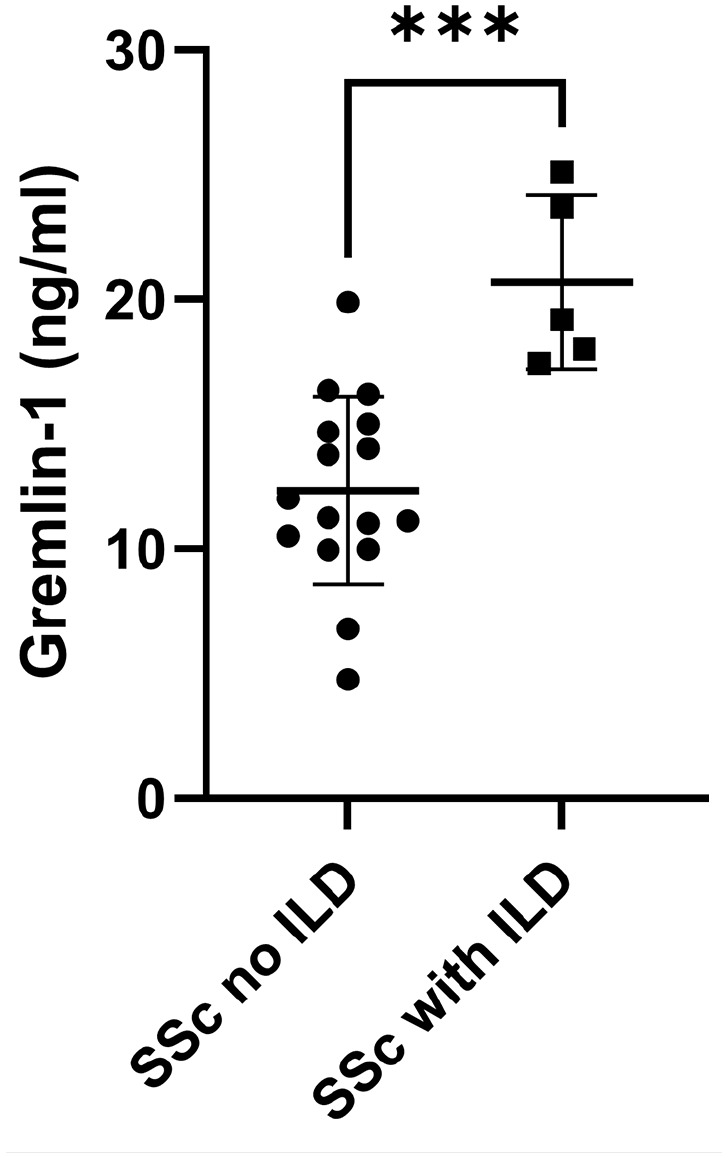
Elevated Gremlin-1 in SSc patients subdivided with those who have and have not ILD. Data are individual points for each donor. ***p = 0.007 (Mann–Whitney *U* test).

Patients with SSc-associated ILD^5^ compared to SSc patients without ILD^12^ had significantly elevated serum Gremlin-1 levels (*p* = 0.0007; Mann–Whitney *U* test; Figure 2).

**Figure 3.**
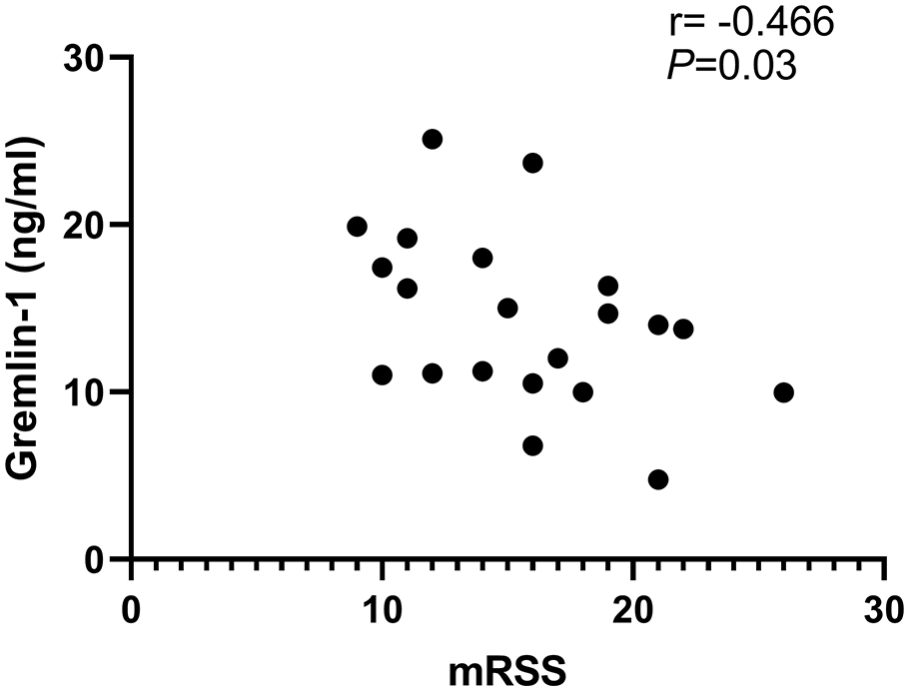
Correlation between Gremlin-1 and mRSS skin score. Negative correlation between mRSS and serum Gremlin-1. *p* = 0.033; *r* = −0.466; *n* = 21 donors.

We next sought to ascertain if there is a correlation between sera Gremlin-1 levels and skin thickness using the modified Rodnan Skin Score (mRSS); a weak negative correlation was found between Gremlin-1 amounts and mRSS (*r* = −0.466; *p* = 0.033, *n* = 21) (see Figure 3). This suggests a weak inverse correlation.

## Discussion

We have recently described a pro-fibrotic role of Gremlin-1 in dermal fibrosis in SSc. The aim of this study was to assess the circulating levels of Gremlin-1 in early diffuse SSc. We identified significantly elevated levels of Gremlin-1 compared to HCs. Furthermore, we also demonstrated significantly elevated levels of Gremlin-1 in SSc-associated ILD compared to patients without ILD.

Gremlin-1 is a BMP antagonist that regulates BMP signalling, but it is associated with certain tumours^13^ including breast cancer,^14^ and fibrosis of the kidney, lungs^9^ and we recently described the skin as a target cell type. Overexpression of Gremlin-1 led to a significant increase in Extra cellular matrix (ECM) and cell migration; however, we did not see elevated levels of Gremlin-1 in the fibroblasts. This study sought to identify is SSc early diffuse patients had elevated Gremlin-1 levels. We found among the 21 SSc patients elevated Gremlin-1, and in the patients subdivided into those with ILD^5^ and those without,12 we could see significantly elevated levels of Gremlin-1. This is suggestive of a biomarker for ILD disease associated with SSc.

Given that SSc-associated ILD is responsible for the high mortality associated with the disease,^15^ this could be of possible clinical utility to differentiate patients. Tissue Gremlin-1 has been associated with IPF,^8^ and in IPF cell, Gremlin-1 has been found to be fibrotic and regulated by microRNA27b.^16^ Furthermore, overexpression of Gremlin-1 in rat lungs by adenoviral vector results in lung fibrosis.^12^ Interestingly, in sarcoidosis, the associated development of fibrosis in these patients was associated with a specific polymorphism in Gremlin-1 gene.^17^ Most recently, although serum levels of Gremlin-1 have been found to be significantly elevated compared to controls and also patients with idiopathic interstitial pneumonia also had higher serum Gremlin-1 levels.^18^ This all suggests that Gremlin-1 could be used a clinical biomarker. We particularly focused on early diffuse SSc patients as we had previously found an induction with IL-6 trans signalling^10^ and with IL-6 being such an important molecule in early disease^19^ we focussed on this. It is now accepted that interventions in early disease are the best possible way to modify disease course.^20^ Interestingly, Gremlin-1 has also been found to be critically involved in hereditary pulmonary hypertension in mice and patients with hereditary pulmonary hypertension.21 It is possible that there is an interrelationship between IL-6 and Gremlin-1 levels. Given that IL-6 appears to regulate Gremlin-1 in vitro,^10^ it maybe that high IL-6 serum correlates with Gremlin-1 levels, although in this study we did not measure IL-6. The patients were early diffuse and thus likely to be much more ‘inflammatory’ subsets so one would predict high IL-6 levels.

We also analysed a possible association with skin thickness (by mRSS) and serum Gremlin-1 levels and found a weak negative correlation (*r* = −0.466). This is a relatively weak correlation, and the modesty likely reflects the number of patients. The biological relevance of this, if any, remains unclear to the authors. In summary, we demonstrate elevated levels of the pro-fibrotic morphogen Gremlin-1 and that this is particularly marked in SSc patients with ILD. Whether Gremlin-1 can predict disease progression is currently unknown and we did not perform a prospective study. This should be examined in prospective follow-up studies to determine if it can predict disease. We recently demonstrated that inhibition of Gremlin-1 with small interfering RNA in fibroblasts retarded collagen deposition,^11^ suggesting that Gremlin-1 is a therapeutic target in this disease.
